# Leaching of liquation-feeding furnace dross as a first step for germanium recovery

**DOI:** 10.1186/s13104-024-06832-6

**Published:** 2024-06-26

**Authors:** Michał Drzazga, Mateusz Ciszewski, Sylwia Kozłowicz, Izabela Maj, Michał Ochmański, Adrian Radoń

**Affiliations:** Łukasiewicz Research Network - Institute of Non-Ferrous Metals, ul. Sowińskiego 5, 44-100 Gliwice, Poland

**Keywords:** Germanium, Dross, Leaching, Sulfuric acid, Oxalic acid

## Abstract

**Objective:**

Germanium, an important component of electronics, is considered by many global economies as a critical raw material. Therefore, investigating its potential new sources is crucial for prospective technology development. This paper presents the investigation results on the leaching of liquation-feeding furnace dross using sulfuric and oxalic acid solutions.

**Results:**

The dross contained mostly zinc (68.0% wt.) but also elevated germanium concentration (0.68% wt.). The influence of temperature, time, initial acid concentration, and liquid-to-solid phase ratio (L:S) was examined. It was found that germanium availability via leaching is limited—maximum leaching yields using aqueous solutions of sulfuric and oxalic acids were 60% (80 °C, 2 h, 15% wt. H_2_SO_4_, L:S 25:1) and 57% (80 °C, 3 h, 12.5% wt. H_2_C_2_O_4_, L:S 10:1), respectively.

## Introduction

The development of modern industry depends on the availability of various limited resources. Due to economic importance and elevated supply risk, some are considered critical raw materials (CRM) and minerals. One example is germanium listed as CRM by major world economies, eg. the USA, EU, Japan, Canada, and Australia [1]. It is associated with its application in fibre optics, infrared optics, and satellite solar cells as well as great dependence on supply from one source [2, 3]. Over 2/3 of its global supply comes from China [4]. Two sources of refined primary germanium are by-products of zinc metallurgy and fly ash from coal combustion, and their share in total output is almost the same [5, 6]. There is still huge room for improvement as less than 3% of germanium contained in zinc ores is recovered nowadays [4, 5].

The recovery process generally starts by identifying the stream in which germanium is concentrated. Examples of materials from zinc metallurgy with elevated germanium concentrations include residues from hydrometallurgical processes obtained during the leaching of zinc ores and purification of zinc electrolytes [7–11]. In pyrometallurgical operations, such by-products as alloy and slag from lead blast furnaces [12, 13], flue dust from flash copper smelting [14], polymetallic alloy from New Jersey process [15] or dust from fuming of leaching residue [16–18] may be generated. In subsequent stages, the Ge-bearing material is firstly leached, then germanium is selectively recovered from the solution by tannin precipitation, and cementation with iron or zinc powder, either by solvent extraction. Obtained germanium concentrate may be then refined to pure metallic germanium or its commercial compounds—GeCl_4_ or GeO_2_ [6, 19, 20].

Leaching of germanium from suitable by-products may be done using different acids. High Ge recovery yields were reported for hydrochloric [8, 21], sulfuric [9, 21–24], acetic [25], or oxalic [26] acid.

Several different Ge-bearing by-products are generated during pyrometallurgical processes, especially during the production of high-grade zinc using the New Jersey process. This paper presents the investigation results on germanium leaching from liquation-feeding furnace dross using sulfuric and oxalic acids. The dross, a by-product of the New Jersey process obtained in a Polish zinc smelter, was investigated.

## Experimental

### Materials

**Fig. 1:**
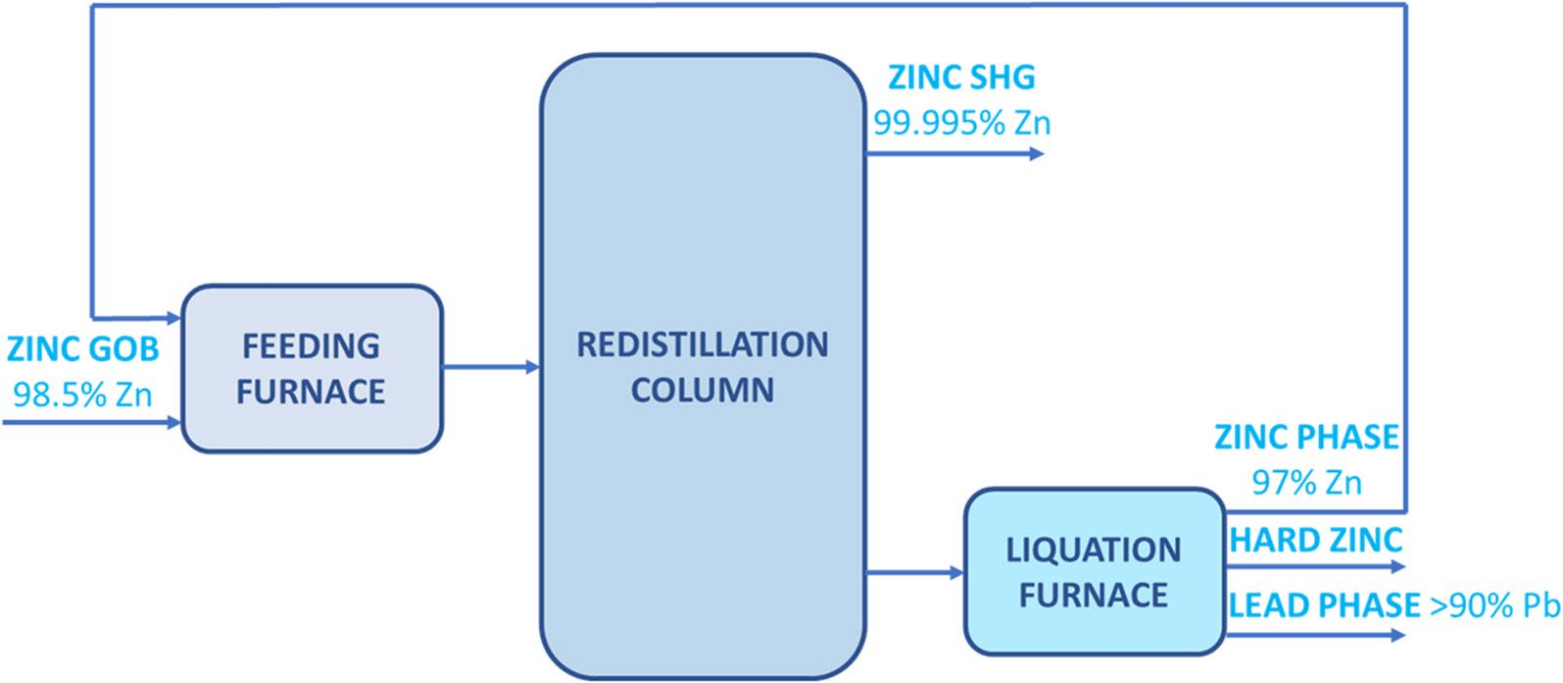
Scheme of New Jersey process (*GOB* Good Ordinary Brand, *SHG* Super High Grade)

The investigated material was liquation-feeding furnace dross collected from a Polish zinc smelter. The furnace is a part of a zinc refining unit using the New Jersey distillation process (Fig. 1). The dross was routinely recycled for the zinc production process. Due to elevated germanium content, the possibility of its recovery was examined in this paper. Large metallic particles were present in the material. Therefore, it was sieved using 0.4 mm mesh and < 0.4 mm fraction was tested.

### Experimental method

Leaching tests were performed using sulfuric acid (98%, Avantor, Poland), oxalic acid (dihydrate, p.a., Chempur, Poland), and hydrogen peroxide solution (30%, Avantor, Poland). The volume of the acid solution was 500 cm^3^ for all trials, while the mass of the dross resulted from the assumed liquid-to-solid ratio (L:S). The influence of temperature, initial acid concentration, L:S, and time (for leaching with oxalic acid) were examined. The leaching time for tests with sulfuric was based on previous experience [27]. Initial acid solutions were prepared by dissolving concentrated acids in deionized water. Then, the pure acid solution was poured into a glass beaker, heated to the desired temperature, and a weighted portion of liquation-feeding furnace dross was added. The suspension was mixed for a set period and then vacuum filtered using a Buchner funnel. In hydrogen peroxide tests, an oxidant was added after 1 h of leaching under non-oxidative conditions.

Gibbs free energies of possible reactions were estimated using HSC Chemistry 9.6.1 software (Metso, Finland) Reaction Equations module. Data for Zn_2_SnO_4_ were found in the literature [28]. Due to the lack of thermodynamic data for ZnSnO_3_, Cu_5_Ge_2_ and Ge(OH)_2_(C_2_O_4_)_2_^2−^ values for respective mixtures, i.e. ZnO·SnO_2_, 5Cu·2Ge and Ge(OH)_4_·2C_2_O_4_^2−^ were assumed.

### Analysis and characterisation

X-ray diffraction (XRD) was used to analyze liquation-feeding furnace dross and leaching residues. XRD patterns were collected using an X-ray diffractometer Rigaku MiniFlex 600 (Rigaku, Tokyo, Japan) with a copper tube Cu Kα (λ = 0.15406 nm), a tube voltage of 40 kV, and a current of 15 mA, using a D/teX Ultra silicon strip detector.

The concentration of germanium and zinc in the solutions was analyzed using ICP-OES (Inductively Coupled Plasma—Optical Emission Spectrometry; Optima 5300 V, PerkinElmer). Solid samples were chemically dissolved before analysis.

Semiquantitative (SQX) analysis of the dross was performed using wavelength-dispersive X-ray fluorescence (WD-XRF) spectrometer Rigaku ZSX Primus (Rigaku, Tokyo, Japan). The sample was pressed into a pellet using boric acid as a matrix.

## Results and discussion

### Analysis of liquation-furnace dross

**Fig. 2:**
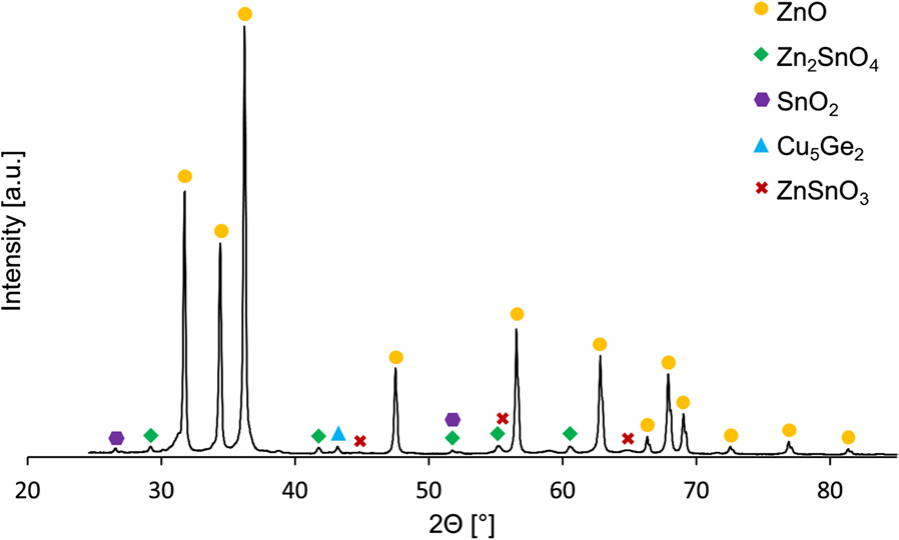
XRD patterns of liquation-feeding furnace dross

Results of the dross chemical and phase analyses are presented in Table 1 and Fig. 2, respectively. The dross predominantly comprised zinc oxide (ZnO), confirmed by chemical and phase analyses. Tin was present mainly in the form of Zn_2_SnO_4_. However, some traces of cassiterite (SnO_2_) and tin zinc oxide (ZnSnO_3_) phases were also indicated. The X-ray diffraction (XRD) pattern analysis showed that the main copper and germanium phase was Cu_5_Ge_2_. It was impossible to determine the lead phase—it might have been an amorphous phase or substitute chemical elements in other phases (for example, Zn in the Zn_2_SnO_4_ phase). Germanium level in the dross (0.68% wt.) was also confirmed by ICP-OES analysis.

**Table 1:** Composition of liquation-feeding furnace dross (SQX analysis)

Element	Zn	O	Sn	Pb	Al	Cu	Ge	Si
Content [% wt.]	68.0	20.1	3.35	2.52	2.22	1.83	0.67	0.25

### Leaching in sulfuric acid

The acid was selected as it was well known that both zinc and germanium reacted with it forming soluble species. On the other hand, tetravalent tin and metallic or monovalent copper did not form soluble species in reaction with sulfuric. It was assumed, also based on available Eh–pH diagrams [29, 30], that the following reactions (Eqs. 1, 2, 3, 4 and 5) were involved during the dross leaching with sulfuric acid under non-oxidative conditions:

1 $${\text{ZnO + H}}_{{2}} {\text{SO}}_{{{4}\left( {{\text{aq}}} \right)}} { } \to {\text{Zn}}^{{2 + }}_{{\left( {{\text{aq}}} \right)}} { + }\,{\text{SO}}_{{4\left( {{\text{aq}}} \right)}}^{2 - } \, + \,{\text{H}}_{{2}} {\text{O}}$$

$$\Delta G_{298}^{0} = - 66.5\,kJ$$

2 $$Zn_{2} SnO_{4} + 2H_{2} SO_{{4_{{\left( {aq} \right)}} }}^{{}} \to 2Zn_{{_{{\left( {aq} \right)}} }}^{2 + } + 2SO_{{4_{{\left( {aq} \right)}} }}^{2 - } + 2H_{2} O + SnO_{2} \downarrow$$

$$\Delta G_{298}^{0} = - 119.7\,kJ$$

3 $$ZnSnO_{3} + H_{2} SO_{{4_{{\left( {aq} \right)}} }}^{{}} \, \to Zn_{{\left( {aq} \right)}}^{2 + } + SO_{{4_{{\left( {aq} \right)}} }}^{2 - } + H_{2} O + SnO_{2} \downarrow$$

$$\Delta G_{298}^{0} \, = \, - 66.5\,kJ$$

4 $$Zn + H_{2} SO_{{4\left( {aq} \right)}} \to Zn_{{\left( {aq} \right)}}^{2 + } + SO_{{4\left( {aq} \right)}}^{2 - } + H_{2} \uparrow$$

$$\Delta G_{298}^{0} = - 147.2\,kJ$$

5 $$Cu_{5} Ge_{2} + 6H_{2} O \to 2H_{2} GeO_{{3\left( {aq} \right)}} + 4H_{2} \uparrow + 5Cu \downarrow$$

$$\Delta G_{298}^{0} = - 70.2\,kJ$$

**Fig. 3:**
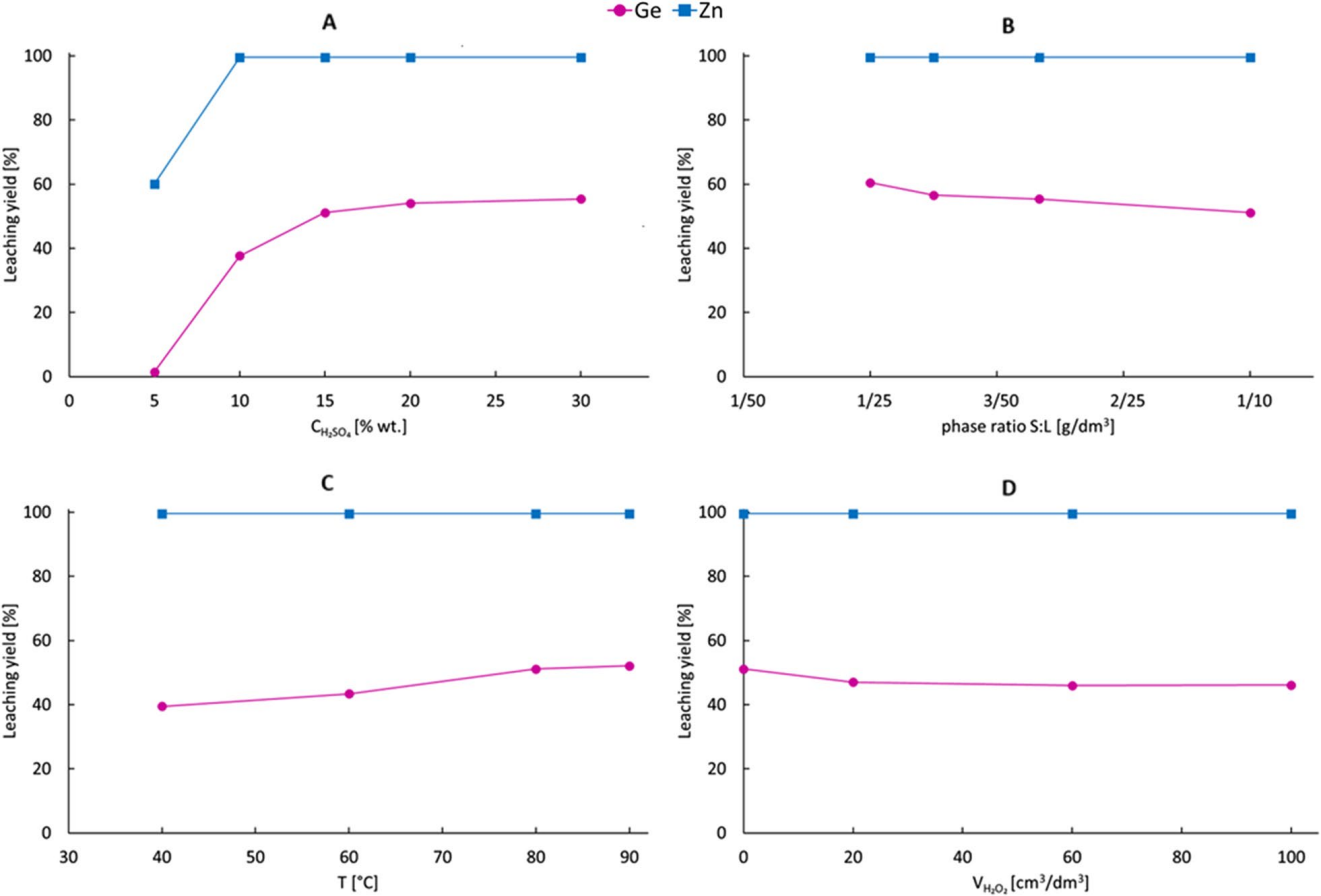
Leaching yields of germanium and zinc from liquation-feeding furnace dross using sulfuric acid under different conditions: **a** influence of initial acid concentration (2 h, 80 °C, L:S 10:1), **b** influence of phase ratio (2 h, 80 °C, 15% wt. H_2_SO_4_), **c** influence of temperature (2 h, 15% wt. H_2_SO_4_, L:S 10:1), **d** influence of 30% H_2_O_2_ dose volume (1 + 1 h after oxidant addition, 80 °C, 15% wt. H_2_SO_4_, L:S 10:1)

**Fig. 4:**
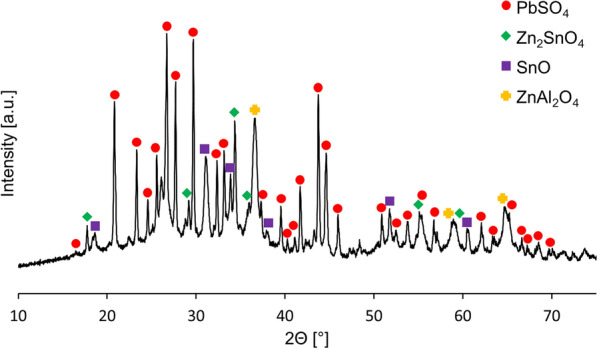
XRD pattern of residue obtained after leaching of liquation-feeding furnace dross in sulfuric acid solution (80 °C, 2 h, L:S 10:1, 15% wt. H_2_SO_4_)

Results of the tests are presented in Fig. 3. XRD analysis for the post-leaching residues is shown in Fig. 4, while its composition is collected in Table 2.

**Table 2:** Composition of residue obtained after leaching of liquation-feeding furnace dross in sulfuric acid (SQX analysis, 80 °C, 2 h, L:S 10:1, 15% wt. H_2_SO_4_)

Element	O	Zn	Sn	Pb	Al	Cu	S	Ge	Si
Content [% wt.]	29.7	18.1	16.2	12.4	10.3	4.75	3.50	2.10	0.42

It was observed that the initial concentration of sulfuric acid influenced the leaching yield of both germanium and zinc. Almost complete zinc leaching was achieved for 10% wt. and higher sulfuric acid concentrations. For 5% wt. H_2_SO_4_ only 60% of zinc was leached. The leaching yield of germanium increased with increasing acid concentration—from < 2% for 5% H_2_SO_4_, through 51% for 15% H_2_SO_4_, to 55% for 30% H_2_SO_4_. It should be highlighted that for acid concentrations > 15% wt. increase of leaching yield was minimal. For low initial concentrations (5% and 10%) of acid, almost all H_2_SO_4_ was consumed, and the final pH was > 2. Therefore, it was not enough acid for complete germanium dissolutions and Ge leaching yields were the lowest. For 15% wt. and higher H_2_SO_4_ concentrations, the final suspension pH was kept between 0 and 1, and due to higher acid availability, the extent of germanium dissolution was higher.

The applied L:S ratio had no influence on zinc leaching yield and minor impact on germanium recovery, which decreased with increasing share of solid phase—from 60% for a 25:1 ratio to 51% for a 10:1 ratio. The final suspension pH was between 0 and 1 for all investigated ratios. A small difference in the yields suggested that incomplete germanium leaching was not limited by its solubility in the water phase. A slight enhancement of germanium leaching yield with increasing temperature was observed—from 39% at 40 °C to 52% at 90 °C. Higher temperatures may have increased the Ge leaching rate, so it was recommended to leach the dross at a temperature ≥ 80 °C.

It was found that the germanium leaching yield under non-oxidizing conditions was not satisfactory—i.e., it was < 61%. One of the reasons might have been that part of germanium was initially present in the copper germanide phase (Cu_5_Ge_2_), which was not easily susceptible to leaching under non-oxidizing conditions. Therefore, tests using hydrogen peroxide as an oxidizer were also performed. It was assumed that copper germanide phase would completely dissolve under oxidative conditions according to the following equations [31]:

6 $$2H_{2} O_{{2\left( {aq} \right)}} \to 2H_{2} O + O_{2\left( g \right)}$$

$$\Delta G_{298}^{0} = - 206.1\,kJ$$

7 $$2Cu_{5} Ge_{2} + 10H_{2} SO_{{4\left( {aq} \right)}} + 9O_{2\left( g \right)} \to 4H_{2} GeO_{{3\left( {aq} \right)}} + 10Cu^{2 + } + 10SO_{{4\left( {aq} \right)}}^{2 - } + 6H_{2} O$$

$$\Delta G_{298}^{0} = - 3771.7\,kJ$$

However, H_2_O_2_ addition had no positive impact on germanium recovery. It was even smaller (45–47%) than in the case of previous tests. Germanium phase was not detected in XRD analysis due to low germanium content (ca. 2.1 wt.%) in the residue. However, an oxidizing agent might have promoted the generation of sparingly soluble germanium dioxide and reduced Ge leaching yield.

Analysis of the residue confirmed that most of the zinc and germanium were leached from the solid phase. Zinc was present as water-insoluble zinc stannate (Zn_2_SnO_4_) [32] and zinc aluminate (ZnAl_2_O_4_) [33]. Moreover, tin was found as insoluble romarchite (SnO) and lead as anglesite (PbSO_4_). The presence of zinc in the residue despite the almost complete Zn leaching related to a high mass loss (> 80%) during the leaching and very high zinc content in the initial materials (68.0%).

### Leaching in oxalic acid

**Fig. 5:**
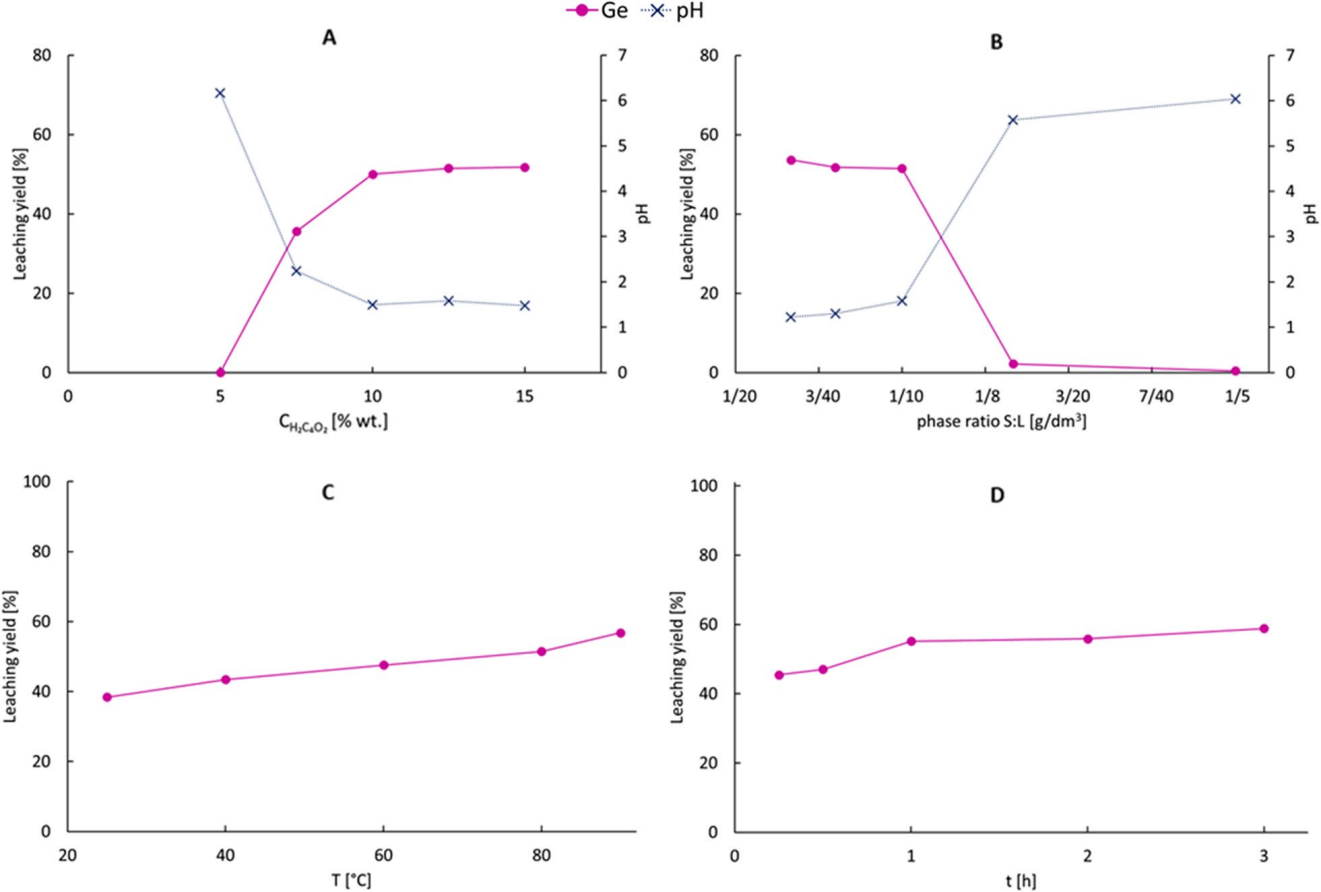
Leaching yields of germanium and zinc from liquation-feeding furnace dross using oxalic acid under different conditions: **a** influence of initial acid concentration (2 h, 80 °C, L:S 10:1), **b** influence of phase ratio (2 h, 80 °C, 12.5% wt. H_2_C_2_O_4_), **c** influence of temperature (2 h, L:S 10:1, 12.5% wt. H_2_C_2_O_4_), **d** influence of time (80 °C, L:S 10:1, 12.5% wt. H_2_C_2_O_4_)

The aim was to find if selective Ge leaching was possible as germanium forms a water-soluble anionic complex with oxalic acid [34], while zinc is precipitated as zinc oxalate [35]. It was also predicted that tin and copper would precipitate as insoluble forms [36, 37]. It was assumed, that the following reactions (Eqs. 8, 9, 10, 11 and 12) were involved during the process. The results of the tests are presented in Fig. 5.

8 $$ZnO + H_{2} C_{2} O_{{4\left( {aq} \right)}} + H_{2} O \to ZnC_{2} O_{4} \cdot 2H2O \downarrow$$

$$\Delta G_{298}^{0} = - 116.4\,kJ$$

9 $$Zn_{2} SnO_{4} + 4H_{2} C_{2} O_{{4\left( {aq} \right)}} \to 2ZnC_{2} O_{4} \cdot 2H_{2} O \downarrow + Sn\left( {C_{2} O_{4} } \right)_{2}$$

$$\Delta G_{298}^{0} = - 166.5\,kJ$$

10 $$ZnSnO_{3} + 3H_{2} C_{2} O_{{4\left( {aq} \right)}} \to ZnC_{2} O_{4} \cdot 2H_{2} O \downarrow + Sn\left( {C_{2} O_{4} } \right)_{2} + H_{2} O$$

$$\Delta G_{298}^{0} = - 63.4\,kJ$$

11 $$Zn + H_{2} C_{2} O_{{4\left( {aq} \right)}} + 2H_{2} O \to ZnC_{2} O_{4} \cdot 2H_{2} O \downarrow + H_{2}$$

$$\Delta G_{298}^{0} = - 197.2\,kJ$$

12 $$Cu_{5} Ge_{2} + 4H_{2} C_{2} O_{{4\left( {aq} \right)}} + 4H_{2} O \to 4H_{{\left( {aq} \right)}}^{ + } + 2Ge\left( {OH} \right)_{2} \left( {C_{2} O_{4} } \right)_{{2\left( {aq} \right)}}^{2 - } + 5Cu \downarrow + 4H_{2} \uparrow$$

$$\Delta G_{298}^{0} = - 35.9\,kJ$$

It was noticed that the germanium leaching yield was increasing as the initial concentration of oxalic acid increased. The highest Ge recovery (52%) was achieved for the highest investigated acid concentration, i.e. 15% wt. It also corresponded with the final pH level, which decreased from 6.2 for 5% H_2_C_2_O_4_ to 1.5 for 15% wt. H_2_C_2_O_4_. Like in the case of leaching using sulfuric acid, when the final suspension pH was < 2, there was not enough acid for a complete reaction with germanium species. The difference in Ge leaching yield between 10% wt. and 12.5% wt. oxalic acid solution was not large—50% and 52%, respectively. However, the larger acid concentration was chosen to ensure the highest extent of the reaction with oxalic acid.

The increase in solid phase share had a negative influence on germanium recovery. It may have been noticed that for L:S ratios between 15:1 and 10:1, Ge leaching yield was 51–54%, while for L:S 7.5:1–5:1, it dropped to < 2%. It corresponded also with the final pH change, which for 15:1–10:1 ratio was 1.2–1.6, while for 7.5:1–5:1–5.6–6.1. Therefore, germanium leaching was greatly influenced by final pH—it dropped below 2% for a final pH > 3.0. These observations were like those observed for H_2_SO_4_ leaching. Too high a final pH was the result of not a sufficient amount of the acid, therefore reducing germanium leaching yield. On the other hand, the leaching yield for L:S ratios 15:1–10:1 was quite similar, suggesting that the non-complete leaching yield was not connected with Ge solubility in the water phase.

The increase in process temperature had a positive impact on germanium leaching yield—38.4% of germanium was leached at 25 °C, while for tests conducted at 90 °C, the Ge leaching yield was 57%. It might have been associated with increased reaction rates associated with the leaching process. An increase in germanium leaching yield was noticed for the first hour of the process—it was 45% after 15 min and 55% after 1 h. Longer reaction times did not result in the enhancement of germanium leaching. However, a reaction time of 2 h was chosen to ensure the highest extent of the reaction.

Although only germanium was leached from the dross, while the other elements almost completely remained in the solid it was found that the concentration of the elements in the leaching residue (Table 3) was lower than in the initial dross (Table 1). The reason was that the mass of residue was > 80% higher than the mass of the dross. It was due to the conversion of the initial phases to oxalates.

**Table 3:** Composition of residue obtained after leaching of liquation-feeding furnace dross in oxalic acid (SQX analysis, 80 °C, 2 h, L:S 10:1, 12.5% wt. H_2_C_2_O_4_)

Element	O	Zn	C	Pb	Al	Cu	Sn	Ge	Si
Content [% wt.]	43.8	38.6	12.6	1.57	1.25	0.89	0.87	0.15	0.08

## Conclusion

Liquation feeding-furnace dross might be an interesting source for germanium recovery. It contains a significant amount of germanium (0.68%). However, its availability via leaching using sulfuric and oxalic acid leaching is quite limited—maximum leaching yields were 60% (80 °C, 2 h, 15% wt. H_2_SO_4_, L:S 25:1) and 57% (80 °C, 3 h, 12.5% wt. H_2_C_2_O_4_, L:S 10:1), respectively. The dross might require preprocessing before leaching as, according to XRD analysis, germanium is primarily bound in the Cu_5_Ge_2_ phase. The preprocessing of dross before leaching may be necessary.

## Limitations

Preliminary results of leaching tests are presented in the study. More detailed studies should also be focused on the preprocessing of dross before leaching to achieve higher germanium leaching yields.

## Data Availability

The datasets generated during and/or analyzed during the current study are available from the corresponding author on reasonable request.
